# Hidden in Plain Sight: Electromechanical Window Negativity in Congenital Long QT Syndrome

**DOI:** 10.1002/ccr3.73336

**Published:** 2026-09-02

**Authors:** Toby Paterson, Roy Sanders, Vivetha Pooranachandran

**Affiliations:** ^1^ Department of Natural Sciences Middlesex University London UK; ^2^ Department of Cardiology North West Anglia Hospital Peterborough UK; ^3^ Department of Cardiology Sheffield Teaching Hospital Sheffield UK

**Keywords:** echocardiography, electromechanical window, implantable cardioverter‐defibrillator, long QT syndrome, ventricular arrhythmia

## Abstract

Risk stratification in congenital long QT syndrome (LQTS) continues to rely heavily on genotype and QTc duration, despite their limited ability to identify individuals at highest arrhythmic risk. The electromechanical window (EMW) is a validated marker of malignant arrhythmia susceptibility, yet its presence within routine echocardiographic imaging has not been recognized. We report a 19‐year‐old woman with *KCNH2*‐related LQTS who experienced recurrent syncope triggered by abrupt auditory stimuli, with ambulatory monitoring revealing self‐terminating torsades de pointes up to 273 beats/min. Although cardiac magnetic resonance and standard echocardiography showed normal structure, retrospective analysis of archived routine cine loops revealed marked EMW negativity (mean −97.5 ms), measurable directly from standard imaging without specialized acquisition. Retrospective analysis demonstrated that marked EMW negativity was measurable from routine echocardiographic images acquired before the diagnosis was established. This case suggests that EMW negativity may be silently present in many previously imaged LQTS patients and recoverable retrospectively from existing echocardiographic datasets.

## Key Clinical Message

EMW negativity may be embedded in everyday echocardiograms and detectable without specialized acquisition.

## 1 Introduction

Congenital long QT syndrome (LQTS) is a heritable channelopathy characterized by delayed myocardial repolarisation and a propensity for malignant ventricular arrhythmias, including torsades de pointes (TdP) and sudden cardiac death [1]. While marked QTc prolongation is associated with a higher risk of arrhythmic events, patients with borderline or equivocal QTc prolongation are not free from risk. Current clinical markers, including QTc duration, genotype, and clinical history, have limited ability to identify the minority of these patients who remain susceptible to life‐threatening ventricular arrhythmias [2].

Experimental and translational studies have demonstrated that LQTS is associated not only with electrical abnormalities but also with altered myocardial mechanics, including prolonged and heterogeneous left ventricular contraction [1]. The electromechanical window (EMW), defined as the difference between the duration of mechanical systole (Q‐onset to aortic valve closure) and the QT interval, integrates these electrical and mechanical phenomena into a single measurable parameter [3]. EMW is normally positive in healthy individuals but becomes negative in LQTS, reflecting a reversal of the normal temporal relationship between repolarisation and mechanical systole [4].

Large cohort studies have shown that EMW negativity is more pronounced in symptomatic genotype‐positive patients and predicts arrhythmic events independently of QTc [4]. Despite growing mechanistic and cohort‐level evidence, EMW assessment has not been incorporated into routine echocardiographic workflows, and its recoverability from standard archived imaging outside prospective research protocols remains largely unexplored. We report a case in which EMW negativity was retrospectively identified from routine echocardiographic recordings in a structurally normal heart preceding multiple malignant ventricular arrhythmias, illustrating that clinically important electromechanical information may be identifiable retrospectively from routine echocardiographic datasets.

## 2 History of Presentation

A 19‐year‐old woman presented with recurrent episodes of transient loss of consciousness. Several episodes occurred immediately upon awakening to an alarm clock. Events were abrupt, without prodromal symptoms such as nausea or visual disturbance, and were followed by spontaneous recovery within minutes. There was no postictal confusion. She reported no preceding chest pain or palpitations. The recurrent nature and association with sudden auditory stimulation raised suspicion of an adrenergically mediated channelopathy.

## 3 Past Medical History

She had no known medical conditions, no prior cardiac investigations, and was not taking regular medications. There was no known family history of syncope, seizures, or sudden cardiac death.

## 4 Differential Diagnosis

The initial differential diagnosis included reflex syncope, epileptic seizure, supraventricular tachycardia with aberrancy, and polymorphic ventricular tachycardia related to a congenital channelopathy.

## 5 Investigations

**FIGURE 1:**
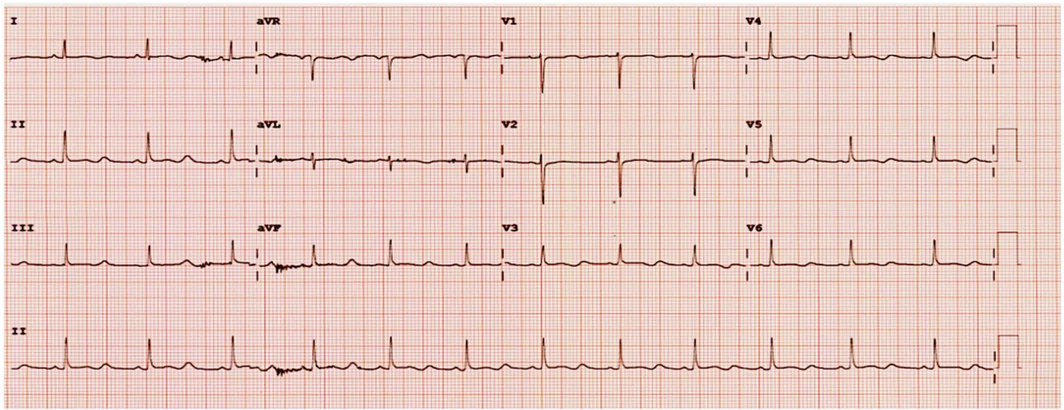
Initial resting 12‐lead ECG demonstrated sinus rhythm with a heart rate of 75 bpm, QRS duration 80 ms, QT interval 480 ms and QTc (Bazett's formula) 537 ms.

**FIGURE 2:**
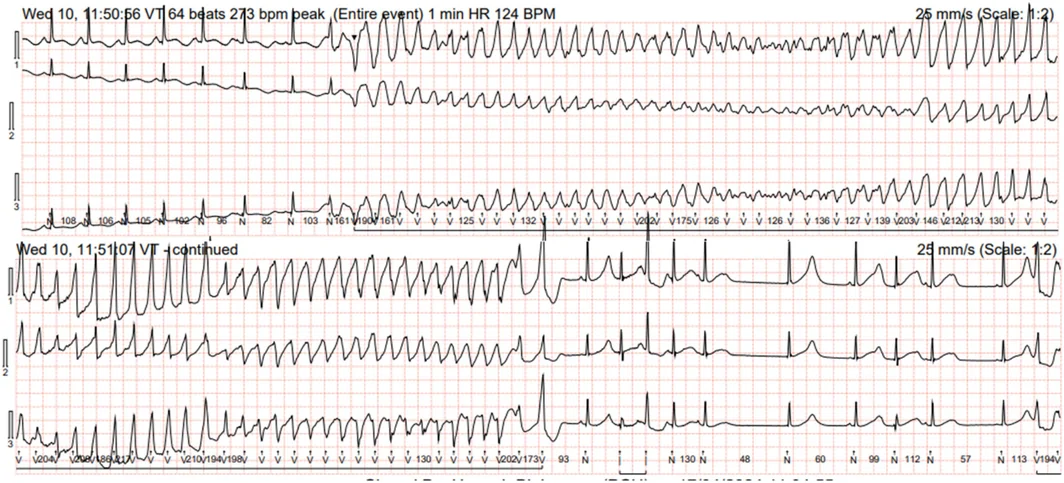
Ambulatory ECG monitoring identified a self‐terminating episode of torsades de pointes lasting 22 s, with a peak ventricular rate of 273 bpm.

**FIGURE 3:**
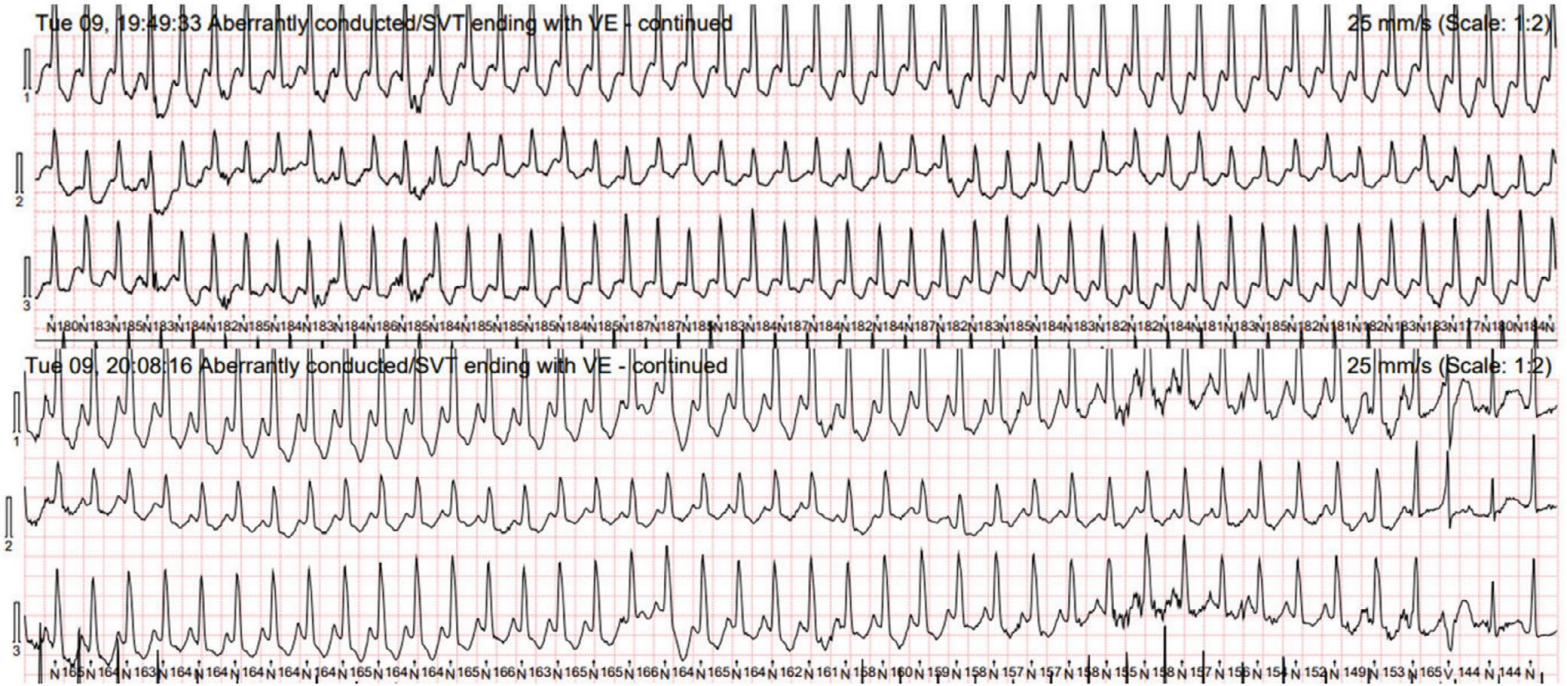
Aberrantly conducted supraventricular tachycardia detected on Ambulatory ECG monitoring lasting 22 min, with a peak ventricular rate of 200 bpm.

A resting ECG made in April demonstrated a prolonged QT interval (Figure 1). Ambulatory ECG (Holter) monitoring identified a self‐terminating run of TdP lasting 22 s with a peak ventricular rate of 273 bpm (Figure 2). In addition, a 22‐min episode of aberrantly conducted supraventricular tachycardia was recorded, with heart rates up to 200 bpm (Figure 3). These findings indicate a malignant ventricular arrhythmic substrate. The symptom diary was not returned, and symptom correlation could not be confirmed.

Structural imaging was performed shortly after the initial TdP episode. Routine transthoracic echocardiography revealed normal chamber dimensions, preserved biventricular systolic function with a left ventricular ejection fraction of 67%, normal diastolic indices, and no valvular pathology. Cardiac magnetic resonance imaging demonstrated normal biventricular size and systolic function. There was no late gadolinium enhancement, myocardial oedema, or structural abnormality to account for the arrhythmia burden.

Blood tests were normal, and genetic testing identified a heterozygous pathogenic missense *KCNH2* c.1838C>T (p.Thr613Met) variant consistent with a high‐risk congenital long QT syndrome.

## 6 Electromechanical Window

EMW was measured retrospectively from archived echocardiographic images and was not available at the time of initial risk stratification. Despite the structurally normal appearance, advanced mechanical assessment demonstrated a profoundly negative EMW. Continuous‐wave Doppler interrogation of the left ventricular outflow tract from the apical long‐axis view was used to measure mechanical systole and the QT interval in lead II.

**FIGURE 4:**
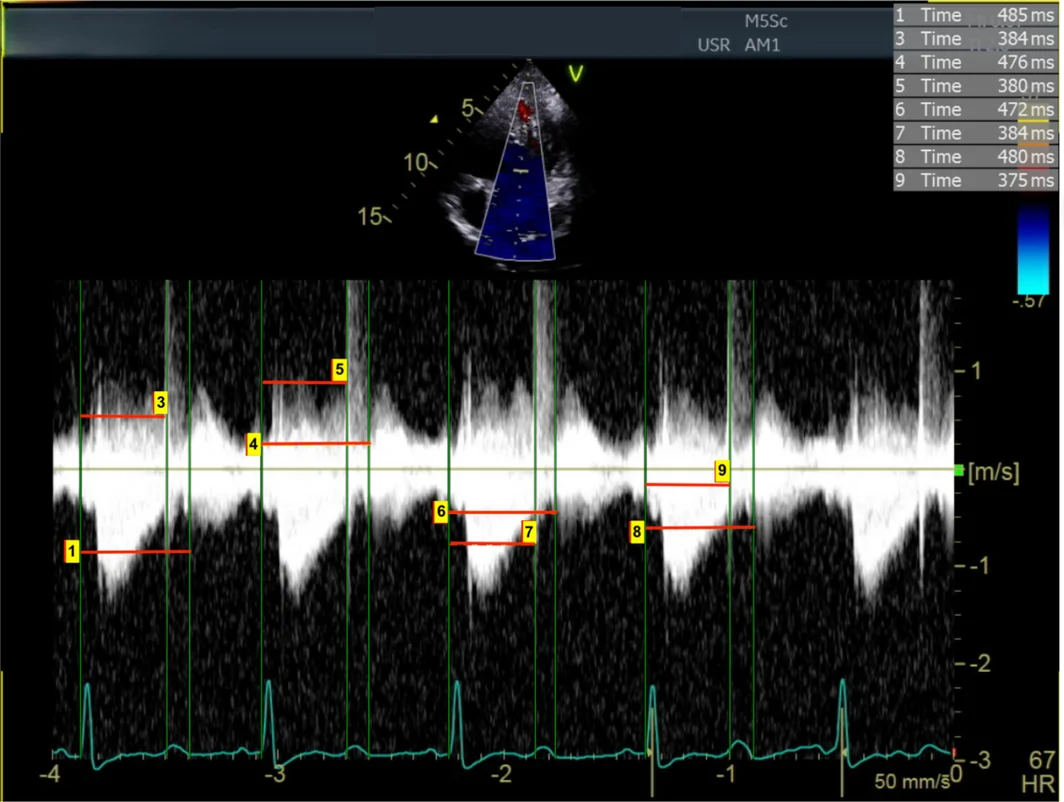
Representative measurement of the electromechanical window (EMW). The green calipers represent timing measurements generated by the echocardiographic software. Measurement 1 corresponds to the QT interval (Q onset to end of T wave; 485 ms), while Measurement 3 corresponds to mechanical systole (Q onset to aortic valve closure; 384 ms). EMW was calculated as mechanical systole minus QT interval (384–485 = −101 ms). Measurements were repeated across multiple cardiac cycles, yielding a mean EMW of −97.5 ms.

EMW was assessed across systoles within the cine loop, yielding values of −101, −96, −88, and −105 ms. A mean EMW of −97.5 ms was calculated, representing a significant inversion of the normal electrical–mechanical relationship (Figure 4). Measurements were independently performed and confirmed by two cardiac technicians.

## 7 Management

Given documented TdP and subsequent ventricular arrhythmia on loop recorder monitoring, a single‐chamber implantable cardioverter‐defibrillator (ICD) was implanted in August 2024. Propranolol and mexiletine were commenced. ICD implantation was undertaken in accordance with the 2022 ESC guideline (Class I recommendation) for survivors of documented ventricular arrhythmia/cardiac arrest or patients with congenital LQTS experiencing recurrent ventricular arrhythmias despite optimal medical therapy [5].

## 8 Outcome and Follow‐Up

The patient received ICD therapy 1 month after implantation. An episode of bradycardia with multifocal PVCs progressed to polymorphic VT at approximately 250 bpm, which was successfully terminated with a single 40 J shock. She remains under specialist surveillance.

## 9 Discussion

Risk stratification in congenital long QT syndrome remains challenging. Conventional approaches based on QTc duration, genotype, clinical history, and composite scoring systems provide population‐level prognostication but have limited ability to identify individuals at imminent risk [6, 7, 8]. The EMW, integrating electrical repolarisation with mechanical systolic duration, offers a more comprehensive assessment of electromechanical coupling [1, 9].

In genotyped cohorts, EMW negativity is more pronounced in symptomatic individuals and independently predicts arrhythmic events, with incremental value over QTc alone [4, 8]. Temporal analyses further suggest that EMW reflects a dynamic state of electromechanical instability, becoming increasingly negative in the period surrounding TdP [4].

In this case, EMW negativity (−97.5 ms) was identified retrospectively from routine echocardiographic recordings, far exceeding values typically reported in high‐risk cohorts (−50 to −70 ms) [4]. Although these measurements were not available at the time of clinical decision‐making, their retrospective analysis demonstrated a degree of inversion that, in the absence of structural heart disease, is consistent with a malignant electromechanical substrate rather than myocardial remodeling. The clinical presentation further supports this interpretation. Syncope triggered by sudden auditory stimuli is characteristic of adrenergically mediated arrhythmia in congenital LQTS. Acute sympathetic activation may exacerbate electromechanical uncoupling, increasing EMW negativity and creating a substrate for triggered ventricular arrhythmia [1].

Importantly, knowledge of the markedly negative EMW would not have altered management in the present case. The patient had recurrent arrhythmic syncope, documented TdP, and a pathogenic *KCNH2* variant, all of which established a high‐risk phenotype warranting aggressive therapy, including ICD implantation. Consequently, EMW would not have changed the therapeutic strategy in this patient. Rather, the potential clinical value of EMW may lie in patients with congenital LQTS whose risk is less clearly defined by conventional markers, where it may provide complementary prognostic information alongside QTc, genotype, and clinical history.

Although current guidelines do not incorporate EMW into routine risk stratification [5, 10, 11], this case demonstrates its practical feasibility. EMW was derived from standard archived echocardiographic cine loops without specialized acquisition or post‐processing, and measurements were reproducible across cardiac cycles and observers. This observation suggests that routine echocardiographic archives may contain clinically relevant electromechanical information that has not previously been assessed during standard reporting. Whether systematic incorporation of EMW into clinical practice improves risk prediction beyond established markers requires prospective validation before routine implementation can be recommended.

## 10 Conclusions

This case illustrates that electromechanical window negativity can be retrospectively detected from routine echocardiographic imaging in congenital long QT syndrome. Larger prospective studies are required to determine its incremental role in clinical risk stratification.

## Author Contributions

**Vivetha Pooranachandran:** conceptualization, writing – original draft, writing – review and editing, supervision. **Toby Paterson:** conceptualization, writing – original draft, formal analysis, data curation, writing – review and editing. **Roy Sanders:** writing – review and editing, formal analysis.

## Funding

The authors have nothing to report.

## Ethics Statement

This study was performed in accordance with the principles of the Declaration of Helsinki.

## Consent

The authors confirm that written consent for the submission and publication of this case, including images, has been obtained from the patient.

## Conflicts of Interest

The authors declare no conflicts of interest.

## Data Availability

The data that support the findings of this study are available on request from the corresponding author. The data are not publicly available due to privacy or ethical restrictions.
